# The Australian Traumatic Brain Injury Initiative: Statement of Working Principles and Rapid Review of Methods to Define Data Dictionaries for Neurological Conditions

**DOI:** 10.1089/neur.2023.0116

**Published:** 2024-04-11

**Authors:** Matthew K. Bagg, Amelia J. Hicks, Sarah C. Hellewell, Jennie L. Ponsford, Natasha A. Lannin, Terence J. O'Brien, Peter A. Cameron, D. Jamie Cooper, Nick Rushworth, Belinda J. Gabbe, Melinda Fitzgerald

**Affiliations:** ^1^ Curtin Health Innovation Research Institute, Faculty of Health Sciences, Curtin University, Bentley, Western Australia, Australia.; ^2^ Perron Institute for Neurological and Translational Science, Nedlands, Western Australia, Australia.; ^3^ Centre for Pain IMPACT, Neuroscience Research Australia, Sydney, New South Wales, Australia.; ^4^ School of Health Sciences, University of Notre Dame Australia, Fremantle, Western Australia, Australia.; ^5^ School of Psychological Sciences, Monash University, Melbourne, Victoria, Australia.; ^6^ Monash-Epworth Rehabilitation Research Centre, Epworth Healthcare, Melbourne, Victoria, Australia.; ^7^ Department of Neuroscience, Central Clinical School, Monash University, Melbourne, Victoria, Australia.; ^8^ Alfred Health, Melbourne, Victoria, Australia.; ^9^ National Trauma Research Institute, Melbourne, Victoria, Australia.; ^10^ School of Public Health and Preventive Medicine, Monash University, Melbourne, Victoria, Australia.; ^11^ Emergency and Trauma Centre, The Alfred Hospital, Melbourne, Victoria, Australia.; ^12^ Department of Intensive Care and Hyperbaric Medicine, The Alfred Hospital, Melbourne, Victoria, Australia.; ^13^ Brain Injury Australia, Sydney, New South Wales, Australia.; ^14^ Health Data Research UK, Swansea University Medical School, Swansea University, Singleton Park, United Kingdom.

**Keywords:** brain injuries, common data elements, neurology, systematic review [publication type], traumatic

## Abstract

The Australian Traumatic Brain Injury Initiative (AUS-TBI) aims to develop a health informatics approach to collect data predictive of outcomes for persons with moderate-severe TBI across Australia. Central to this approach is a data dictionary; however, no systematic reviews of methods to define and develop data dictionaries exist to-date. This rapid systematic review aimed to identify and characterize methods for designing data dictionaries to collect outcomes or variables in persons with neurological conditions. Database searches were conducted from inception through October 2021. Records were screened in two stages against set criteria to identify methods to define data dictionaries for neurological conditions (International Classification of Diseases, 11th Revision: 08, 22, and 23). Standardized data were extracted. Processes were checked at each stage by independent review of a random 25% of records. Consensus was reached through discussion where necessary. Thirty-nine initiatives were identified across 29 neurological conditions. No single established or recommended method for defining a data dictionary was identified. Nine initiatives conducted systematic reviews to collate information before implementing a consensus process. Thirty-seven initiatives consulted with end-users. Methods of consultation were “roundtable” discussion (*n* = 30); with facilitation (*n* = 16); that was iterative (*n* = 27); and frequently conducted in-person (*n* = 27). Researcher stakeholders were involved in all initiatives and clinicians in 25. Importantly, only six initiatives involved persons with lived experience of TBI and four involved carers. Methods for defining data dictionaries were variable and reporting is sparse. Our findings are instructive for AUS-TBI and can be used to further development of methods for defining data dictionaries.

## Introduction

Traumatic brain injury (TBI) is a widespread, burdensome, and costly health experience, which is estimated to impact 55 million persons each year, globally.^1^ Australians and New Zealanders are estimated to experience from 46 through 790 TBIs per 100,000 persons, which extrapolates to ∼200,000 moderate-severe injuries in Australia each year.^1–3^ Moderate-severe TBI can be a devastating experience from which many people do not survive.^4–6^ Similarly devastating is the potential lifetime experience of disability; manifest in various respects for the injured person, their family, and society more broadly.^7–16^ Although impersonal indices, the estimated annual addition of $AU2 billion in treatment-related costs, and a global economic cost of $US400 billion, highlight the scale of the challenge.^7^

There are vigorous global efforts underway to meet this challenge.^17–21^ Yet, innovation is warranted because outcomes do not appear to be improving.^22,23^ The complexity of the TBI experience may be fundamental here. The impacts of TBI are influenced by a confluence of experiences across at least five domains—social and other health factors, clinical and treatment-related factors, and biological mechanisms—that interact to manifest in a broad range of possible outcomes. This complexity may underpin the ongoing difficulty in accurate prognostication and obstruct understanding of contributing factors that is needed to optimize treatment.^23–28^

The Australian Traumatic Brain Injury Initiative (AUS-TBI) is a collaboration of clinicians, scientists, and persons with lived experience seeking to improve prediction, healthcare, and outcomes for persons experiencing moderate-severe TBI.^29^ AUS-TBI is designing a coherent, federated, health informatics approach for the collection, management, and use of information about moderate-severe TBI in Australia. This approach is intended to facilitate improved understanding of the TBI experience for use in outcome prediction^30,31^ and treatment evaluation.^32,33^ For example, the approach will facilitate application of novel analyses to improved data sources to build clinical prediction tools. Central to the viability of the approach in meeting these aims is a comprehensive evidence-based, practice-informed, data dictionary. Data dictionaries are a set of common data elements (CDEs)—units of data with well-defined attributes—that constitute the ontology for a coherent data structure.^19,32,34,35^

The success of AUS-TBI will be founded, in part, on robust methods for designing the required data dictionary. Although data dictionaries have been defined in TBI,^36–40^ at the commencement of AUS-TBI there were no known systematic reviews examining the methods, or best-practice guidelines, used to develop data dictionaries for TBI, although guidance exists for other neurological conditions^41–44^ and in other fields.^45–48^ In-depth knowledge of what has been done in past initiatives to design data dictionaries across neurological conditions may be instructive for AUS-TBI.

The aims of the rapid systematic review reported in this article were to identify and characterize the initiatives themselves, and the methods used, to design data dictionaries for neurological conditions. After the systematic review, this article reports the methods subsequently chosen for AUS-TBI and introduces the thematic study areas through which the data dictionary was compiled. Accompanying articles in this series report the further activities in the six study areas,^29^ with a summative report of the AUS-TBI data dictionary in the final article.

## Methods

The rapid systematic review followed a protocol submitted for registration on PROSPERO on November 27, 2021 and lodged the same day on the Open Science Framework.^49^

### Objectives

The objectives of the review were to: 1) systematically identify records of initiatives to design data dictionaries for neurological conditions and 2) characterize the methods used in each initiative.

### Sampling

Standardized strategies were used to search MEDLINE, Embase, Emcare, PsycINFO (through Ovid), and CINAHL (through EBSCOHost) from inception through November 27, 2021. Records were managed in *Zotero*^50^ and deduplicated and screened in *Rayyan*.^51^ All records were screened against the sampling criteria in two stages: 1) all records based on title and abstract alone and 2) the full length of all potentially eligible records. Records were included if they were a full-length, English-language, peer-reviewed journal article describing any form of consensus-making process for designing data dictionaries for any human neurological condition. Consensus-making processes were considered inclusive of consensus, agreement, concurrence, majority opinion, or similar being sought from stakeholders. Records describing processes that did not explicitly refer to consensus among stakeholders (or the above synonyms) were excluded. Data dictionaries/CDEs were considered inclusive of data elements, biomarkers, data standards, data items, and outcome measures. Neurological conditions listed under set 08 “Diseases of the Nervous System” in the International Classification of Diseases, 11th Revision (ICD-11)^52^ were considered. Neurological conditions listed under sets 22 “Injury, poisoning or certain other consequences of external causes” and 23 “External causes of morbidity or mortality” were also considered. A single reviewer screened all records, and a second independently screened random sets of 25% (of records) at either stage. Consensus was achieved through discussion where necessary.

Included records were linked to discern unique initiatives to design data dictionaries. A single reviewer used the available information in study abstracts, methods, and reference sections to identify whether the same initiative was referenced in multiple records. Linked records were grouped under the most recently published record (the primary record). Where an individual record was not linked, it was presumed to be the sole and primary record for the relevant initiative. These judgements were cross-checked independently by a second reviewer.

### Measurement and appraisal

Data were extracted from the primary record for each initiative using a standardized *Microsoft Excel*^54^ sheet that was pilot tested on three records. The extracted data items included bibliographic details of the record, commissioning and funding organizations, the neurological condition, details of the consensus-making process (including type, iterations, stakeholders, conveners, communication, and definition of consensus), and details of the data elements (including their source [e.g., from literature searches], novelty, and output). A second reviewer independently checked data from a random 25% of records. Consensus was achieved through discussion where necessary. Data were read to R software,^55^ summarized for description using the tidyverse,^56^ readxl,^57^ and compare^58^ and written to tables for presentation.

## Results

The search identified 2599 records, of which 982 were duplicates. A total of 1617 records were screened. A total of 1422 records were excluded based on title and abstract, and the full length procured for the remaining 195 potentially eligible records. One hundred twenty-one full-length records were excluded, leaving 74 records included in this iteration of the review (Supplementary Data). Random sets were screened independently by a second reviewer at either stage, with complete agreement at title/abstract stage. Agreement for full-length screening was incalculable because of an error with export from the screening software. Fifteen records were excluded during data extraction, with 0.8 (12 of 15) initial agreement and complete final agreement between reviewers.

**FIG. 1.**
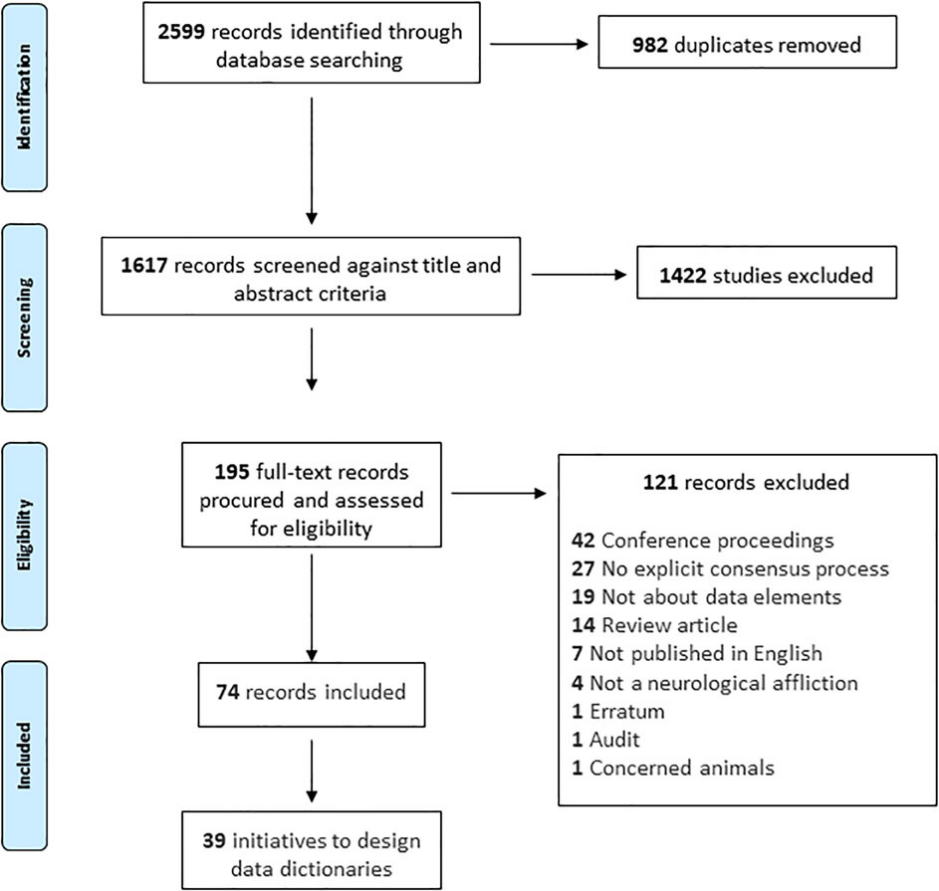
Search and screening process.

The 74 included records reflected reports of 39 unique initiatives to define data dictionaries across 29 different neurological conditions. These are presented in Table 1. The most common condition was Alzheimer's disease (6 of 29; 20.7%). Multiple sclerosis, Parkinson's disease, dementia (not otherwise classified), neuroendocrine neoplasia, and mild cognitive impairment were each the subject of two initiatives (2 of 29; 6.7%). There was a single initiative for adult TBI, two for pediatric TBI, and one each for adult and pediatric spinal cord injury. A further two initiatives were for explicitly labeled pediatric conditions: epilepsy and high-grade glioma. The names of the initiatives, where identified, are listed alongside the conditions in Table 1. The most common initiatives were the National Institute of Neurological Disorders and Stroke (NINDS) CDE Projects. Reports of the 39 initiatives have been published annually from 2011 through 2021, as well as in 1998, 2003, and 2009.

**Table 1.** Descriptions of and Outputs From Initiatives to Define Data Dictionaries

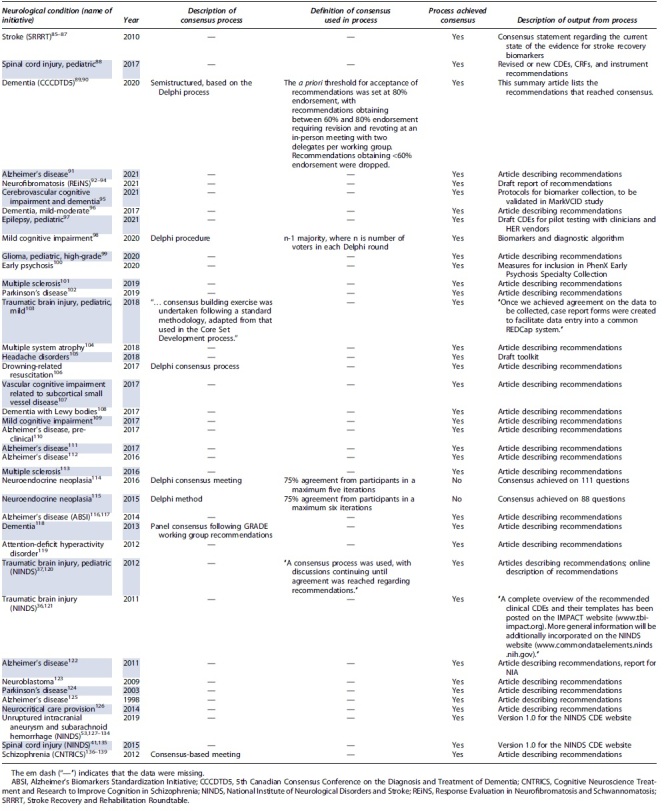

An explicit description or name for the consensus process was identified for eight initiatives (8 of 39; 20.5%) and missing otherwise. Five of these named processes referenced the Delphi methodologies (5 of 39; 12.8%). An explicit definition of what consensus meant (with respect to the process) was identified for five (5 of 39; 12.8%) initiatives and missing otherwise. Four of these definitions included quantitative statements (Table 1). The fifth stated that “… discussions [continued] until agreement was reached…”.^37^

Thirty-seven processes reported achieving consensus on the set of data elements (37 of 39; 94.9%). Two initiatives reported achieving consensus on a subset of data elements (2 of 39; 5.1%). The various products from the consensus processes are described in Table 1. Notably, the majority of initiatives produced a published article describing the CDEs for which consensus was achieved. A minority of initiatives reported application of the CDEs in case-report forms, registry design, and biomarker collection.

All initiatives involved multiple stakeholders (presumed researchers unless otherwise stated). Twenty-five initiatives explicitly involved clinicians as stakeholders in the consensus process, six initiatives involved persons with lived experience and four involved carers of persons with lived experience (25 of 39 [64.1%], 6 of 39 [15.4%], and 4 of 39 [10.3%], respectively). The selection of stakeholders was explicitly described for 18 consensus processes (18 of 39; 46.2%) and are reported verbatim in Table 2.

**Table 2.** Stakeholder Involvement in Consensus Processes to Define Data Dictionaries

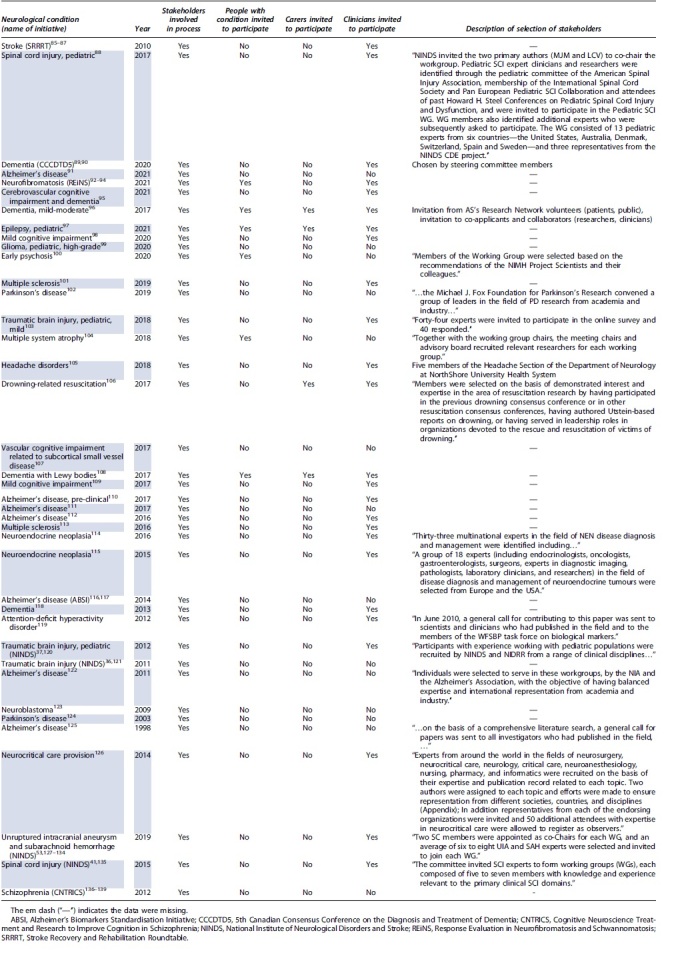

The reported initiatives used various methods of communication (Table 3). The processes were facilitated in 16 instances (16 of 39; 41.0%); by so-termed chairs, moderators or leaders of the initiative, or derivative working groups. Fifteen consensus processes involved derivative groups, and 30 involved roundtable discussion (15 of 39 [38.5%], 30 of 39 [76.9%]). Twenty-seven consensus processes involved iterations (27 of 39; 69.2%), which ranged from at least one to at least 10. Stakeholders communicated through face-to-face meetings (27 of 39; 69.2%), telephone calls (8 of 39; 20.5%), and electronic correspondence (15 of 39; 38.5%). A single consensus process used a password-protected website for collection and display of feedback.

**Table 3.** Communication Used in Consensus Processes to Define Data Dictionaries

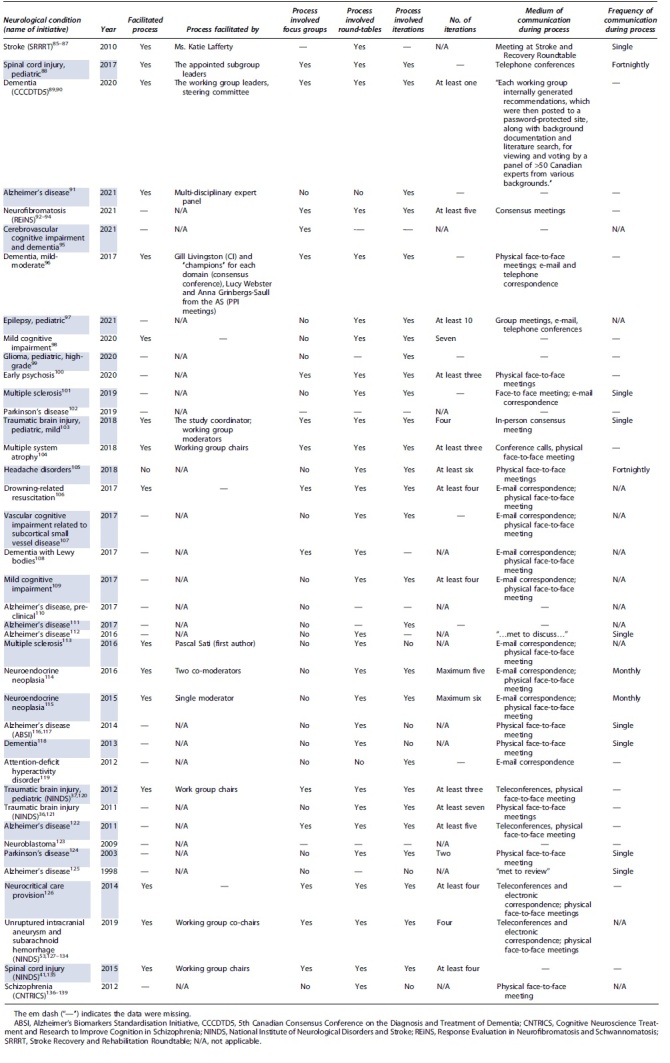

The reported initiatives used various sources of information during the consensus process (Table 4). In the majority, stakeholders used extant data dictionaries or individual data elements in the consensus process (21 of 39 [53.8%] and 25 of 39 [64.1%], respectively). Novel data elements were generated in two instances (2 of 39; 5.1%). The extant data elements were identified through systematic reviews (9 of 39; 23.1%), narrative reviews, or existing data dictionaries. Eight initiatives conducted systematic reviews *de novo* for the purpose of the initiative.

**Table 4.** Information Sources Used in Consensus Processes to Define Data Dictionaries

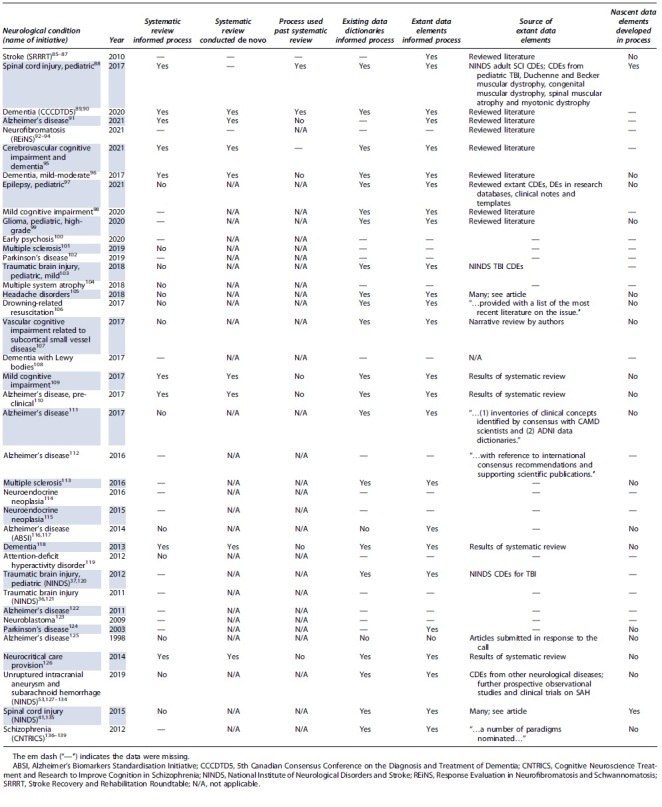

### Differences between the protocol and this iteration of the review

The protocol had stated that a main outcome of the review was to establish the number of unique available methods for defining CDEs. This was removed because, on reflection, the objectives of the review did not map to outcomes in the conventional sense. We chose to specify no outcomes because whereas a single outcome was overly reductive, neither did multiple outcomes appropriately reflect the data items captured. Second, the search of government, institutional, and consumer sources was not completed for reasons of feasibility and yield. Initial work revealed a yield of 0.032 (14 of 431 titles), which was judged insufficient to merit the available resources. Third, study records were not screened or extracted in duplicate. Rather, a second independent reviewer cross-checked a random sample of records. This established method was selected to improve efficiency, while retaining oversight, to meet required timelines.

## Discussion

This rapid systematic review identified 39 initiatives to define data dictionaries for persons with neurological conditions. Within these initiatives, no single established or best-practice method for defining a data dictionary was identified. Explicit descriptions of the processes were missing in 80% of the reported initiatives. We did not identify any established methods for preliminary work (e.g., identification of candidate data elements, selection of stakeholders), establishing consensus, or communication of the finished products. Rather, the reported methods were variable. The nature of preliminary work and the types of stakeholders involved in the processes varied. Only nine initiatives conducted systematic reviews to collate information before the consensus process, and just two reported using information from past systematic reviews. Several references to the Delphi methodologies were noted, which cover a proportion of the consensus process, namely the consensus deliberations themselves. Notably, nearly all (37 of 39) initiatives reported achieving consensus. On that basis, the breadth of findings across the initiatives are instructive of diverse activities that, in the absence of an established method, may nonetheless successfully yield consensus on a set of CDEs.

Most striking are the definitions of consensus; none were the same. The implication is that arbitrary definitions are not a barrier to achieving consensus. A definition of consensus was only explicitly reported in six instances. Yet, it seems likely that the non-reported definitions would vary, given the absence of an established method. It is not clear whether the lack of an established method influences the likelihood of achieving consensus; however, it is reasonable to presume that the way various viewpoints are integrated will vary if consensus procedures are undefined and/or variable. The reported definitions, at least, were strict and clear, which may have been propitious to consensus.

Notably, too, the way the consensus processes were conducted varied in multiple respects. For example, facilitation of the process, use of derivative groups, and number of iterations were varied. There was consistency of observations among the media of communication used for the consensus processes. It was common for clinicians to be included in consensus endeavors (25 of 39), although persons with lived experience (6 of 39) or their carers (4 of 39) were infrequently involved. The implication is that each of these methodological activities might be used to successfully establish consensus on a data dictionary. However, each of these suppositions require substantiation through methodological work. Indeed, systematic evidence synthesis and widespread stakeholder involvement are increasingly considered the best-practice approach to these activities.^59–78^ The incomplete use of these approaches amidst near-complete consensus across initiatives is further testament to the need for further methodological work.

The number and frequency of initiatives and corresponding neurological conditions identified in this review indicate a strong tendency to establish data dictionaries in this broad field. However, the general nature of reporting warrants highlighting. Numerous data items for this study were missing from the identified reports. This may reflect a low level of disclosure, or a lack of clarity on what needs to be reported in such a record; the latter seems more likely in the absence of established guidance.

Reporting is distinct from, yet often the only index of, the conduct of a study. Thus, the results of this study may have implications for the conduct of further initiatives to establish consensus on CDEs. It is important to interpret the data collated here with the view that this study aimed to identify and characterize what was done, not whether any such thing should have been done. In the future, guidance on methods and reporting of initiatives to design data dictionaries might be sought from empirical evaluations and perhaps from construction of formal guidelines.

This study has several limitations. First, data extracted from the primary record for each initiative may not be representative of the other linked records. Second, the stated neurological conditions were taken directly from the sampled records without consolidation of possible synonyms. Third, conditions listed within subset 6 of ICD-11 might be considered neurological yet were not included in this study. Fourth, we did not search outside the published scientific literature, or in clinical fields other than neurology. Fifth, the search may have missed some initiatives^38,39^ or key records. Collectively, these limitations mean that the data collated here are not an exhaustive set, are subject to change, and may not generalize accurately in other uses. The objectives of this study were descriptive, however, and interpretations were made in respect of these limitations—both in this article and for the Initiative's broader planning. This review will be updated and converted to a “living” synthesis given that this will improve and maintain the accuracy and currency of the collated data.^79,80^

This study has notable strengths. It was prospectively registered, searched numerous sources, and was completed on schedule to inform AUS-TBI. Rapid reviews use established methods from conventional systematic reviews to facilitate rigor.^81–84^ Agreement between reviewers for both screening and data extraction was high. The data set and search strategies are provided in the Supplementary Data, ensuring transparency.

This study has been instructive for AUS-TBI. The methodological features (for defining a data dictionary) identified here have served as an empirical foundation for decision making on how AUS-TBI has designed the process for generating a single data dictionary for the health informatics approach. Three clear examples are the conduct of systematic reviews *de novo*, the overt intent to include a variety of stakeholders, and the commitment to iterative, independently facilitated, consensus processes.

The AUS-TBI Initiative will consider each domain of the TBI experience as a separate study area.^29^ The study areas are: 1) demographic, injury event, and social characteristics; 2) pre-existing health conditions; 3) the clinical experience; 4) biological mechanisms; 5) acute interventions; and 6) long-term outcomes. Methods within each study area have overall coherence under the Initiative and vary to meet the requirements of the scientific, clinical, and consumer contexts of each domain.

To the best of our knowledge, at the outset of AUS-TBI, there was a large and growing literature across each domain that was not satisfactorily captured by extant systematic reviews. Although we identified a lesser number of past initiatives that used systematic reviews to generate the background information needed to generate a data dictionary, the contemporary expectation is that these studies are the ideal type for capture and synthesis of information and thus will be used for AUS-TBI. The potential for a living evidence approach to maintain currency of the reviews amidst a growing literature is an additional factor in the choice to conduct bespoke systematic reviews for AUS-TBI.

It was clear from the results of this study that participation in consensus processes has been limited for persons with lived experience and their carers (Table 2). The AUS-TBI consensus process will incorporate participation from stakeholders with lived experience, carers, clinicians, and researchers. This is in line with contemporary expectations for collaborative design and reflects the pragmatic intent of the Initiative to develop a data dictionary (and subsequent data management and usage approaches) that adequately addresses the aims of the Initiative.^29^

It was frequently reported that consensus processes involved iterations, and slightly less than half were facilitated. AUS-TBI will use an independent facilitator to conduct the consensus meetings, process the findings, and coordinate iterations. This respects the emergent nature of consensus, and the neutral position needed to integrate a plurality of views.

This article has introduced AUS-TBI, reported a rapid review of methods for defining data dictionaries, and noted how this will inform the conduct of AUS-TBI. The following articles in this series report on the use of the method to guide the activities in each of the six study areas, with a summative report of the data dictionary in the final article.

## Supplementary Material

Supplemental data

Supplemental data

## Transparency, Rigor, and Reproducibility Summary

This study was conducted after a planned protocol.^49^ Deviations from protocol have been clearly reported herein. The search strategies and the data set are available with the Supplementary Material to this article.

Annotated R scripts are available on request, subject to written agreement. Further information is available at the discretion of the corresponding author.

## Australian Traumatic Brain Injury Investigators

*Tara Alexander*, Australasian Rehabilitation Outcomes Centre & Australian Health Services Research Institute, Faculty of Business and Law, University of Wollongong, Wollongong, New South Wales, Australia; *Vicki Anderson*, Psychology Service, The Royal Children's Hospital, Melbourne, Victoria, Australia; Clinical Sciences Research, Murdoch Children's Research Institute, Melbourne, Victoria, Australia; *Ana Antonic-Baker*, Department of Neuroscience, Central Clinical School, Monash University, Melbourne, Victoria, Australia; *Elizabeth Armstrong*, School of Medical and Health Sciences, Edith Cowan University, Perth, Western Australia, Australia; *Franz E Babl*, Department of Emergency Medicine, The Royal Children's Hospital, Melbourne, Victoria, Australia; Departments of Paediatrics and Critical Care, University of Melbourne, Melbourne, Victoria, Australia; Murdoch Children's Research Institute, Melbourne, Victoria, Australia; *Matthew K Bagg*, Curtin Health Innovation Research Institute, Faculty of Health Sciences, Curtin University, Perth, Western Australia, Australia; School of Health Sciences, University of Notre Dame Australia, Perth, Western Australia, Australia; Perron Institute for Neurological and Translational Science, Perth, Western Australia, Australia; Centre for Pain IMPACT, Neuroscience Research Australia, Sydney, New South Wales, Australia; *Zsolt J Balogh*, Department of Traumatology, John Hunter Hospital and University of Newcastle, Newcastle, New South Wales Australia; *Karen M Barlow*, Acquired Brain Injury in Children Research Program, Queensland Children's Hospital, Brisbane, Queensland, Australia; Centre for Children's Health Research, University of Queensland, Brisbane, Queensland, Australia; *Judith Bellapart*, Department of Intensive Care Services, Royal Brisbane and Women's Hospital, Brisbane, Queensland, Australia; Faculty of Medicine, University of Queensland, Brisbane, Queensland, Australia; *Niranjan Bidargaddi*, Flinders Digital Health Centre, College of Medicine & Public Health, Flinders University, Adelaide, South Australia, Australia; *Erika Bosio*, Centre for Clinical Research in Emergency Medicine, Harry Perkins Institute of Medical Research, Perth, Western Australia, Australia; School of Biomedical Science & School of Medicine, University of Western Australia, Perth, Western Australia, Australia; *Peter Bragge*, BehaviourWorks Australia, Monash Sustainable Development Institute, Monash University, Melbourne, Victoria, Australia; *Michael Bynevelt*, School of Surgery, The University of Western Australia, Perth, Western Australia, Australia; Neurological Intervention and Imaging Service of Western Australia, Sir Charles Gairdner Hospital, Perth, Western Australia, Australia; *Karen Caeyenberghs*, Cognitive Neuroscience Unit, School of Psychology, Deakin University, Geelong, Victoria, Australia; *Peter A Cameron*, National Trauma Research Institute, Melbourne, Victoria, Australia; School of Public Health and Preventive Medicine, Monash University, Melbourne, Victoria, Australia; Emergency and Trauma Centre, The Alfred Hospital, Melbourne, Victoria, Australia; *Jacquelin Capell*, Australasian Rehabilitation Outcomes Centre & Australian Health Services Research Institute, Faculty of Business and Law, University of Wollongong, Wollongong, New South Wales, Australia; *Kevin E K Chai*, School of Population Health, Faculty of Health Sciences, Curtin University, Perth, Western Australia, Australia; Curtin Institute for Computation, Curtin University, Perth, Western Australia, Australia; *Lyndsey E Collins-Praino*, School of Biomedicine, University of Adelaide, Adelaide, South Australia, Australia; *D J Jamie Cooper*, Australian and New Zealand Intensive Care Research Centre, School of Public Health and Preventive Medicine, Monash University, Melbourne, Victoria, Australia; Department of Intensive Care and Hyperbaric Medicine, The Alfred, Melbourne, Victoria, Australia; *Gill Cowen*, School of Medicine, Faculty of Health Sciences, Curtin University, Perth, Western Australia, Australia; *Louise M Crowe*, Clinical Sciences Research, Murdoch Children's Research Institute, Melbourne, Victoria, Australia; Department of Paediatrics, University of Melbourne, Melbourne, Victoria, Australia; *Tim Cudmore*, AUS-TBI Lived Experience Advisory Group; *Jennifer Cullen*, Synapse, Brisbane, Queensland, Australia; James Cook University, Townsville, Queensland, Australia; Menzies Health Institute Queensland, Griffith University, Brisbane, Queensland, Australia; *Kate Curtis*, Susan Wakil School of Nursing and Midwifery, Faculty of Medicine and Health, The University of Sydney, New South Wales, Australia; Illawarra Shoalhaven Local Health District, Wollongong, New South Wales, Australia; Illawarra Health and Medical Research Institute, Wollongong, New South Wales, Australia; George Institute for Global Health, Sydney, New South Wales, Australia; *Anthony Delaney*, Division of Critical Care, The George Institute for Global Health, Sydney, New South Wales, Australia; Malcolm Fisher Department of Intensive Care Medicine, Royal North Shore Hospital, Sydney, New South Wales, Australia; Northern Clinical School, Sydney Medical School, University of Sydney, Sydney, New South Wales, Australia; Australian and New Zealand Intensive Care Research Centre, School of Public Health and Preventive Medicine, Monash University, Melbourne, Victoria Australia; *Graeme Dibdin*, AUS-TBI Lived Experience Advisory Group; *Sandra Eades*, Centre for Epidemiology and Biostatistics, Melbourne School of Population and Global Health, University of Melbourne, Melbourne, Victoria, Australia; School of Medicine, Faculty of Health Sciences, Curtin University, Perth, Western Australia, Australia; *Gary F Egan*, Monash Biomedical Imaging & School of Psychological Sciences, Monash University, Melbourne, Victoria, Australia; *Daniel Y Ellis*, Department of Trauma, Royal Adelaide Hospital, Adelaide, South Australia, Australia; Statewide South Australian Trauma Service, South Australia, Australia; School of Public Health and Tropical Medicine, James Cook University, Queensland, Australia; *Ari Ercole*, Division of Anaesthesia, University of Cambridge, Addenbrooke's Hospital, Cambridge, United Kingdom; Cambridge Centre for AI in Medicine, University of Cambridge, United Kingdom; *Daniel M Fatovich*, Emergency Medicine, Royal Perth Hospital, University of Western Australia, Perth, Western Australia, Australia; Centre for Clinical Research in Emergency Medicine, Harry Perkins Institute of Medical Research, Perth, Western Australia, Australia; *Murray J Fisher*, Susan Wakil School of Nursing and Midwifery, Faculty of Medicine and Health, University of Sydney, New South Wales, Australia; Royal Rehab, Ryde, Sydney, New South Wales, Australia; *Mark Fitzgerald*, National Trauma Research Institute, Melbourne, Victoria, Australia; *Melinda Fitzgerald*, Curtin Health Innovation Research Institute, Faculty of Health Sciences, Curtin University, Perth, Western Australia, Australia; Perron Institute for Neurological and Translational Science, Perth, Western Australia, Australia; *Jennifer Fleming*, School of Health and Rehabilitation Sciences, The University of Queensland, Brisbane, Queensland, Australia; *Roslyn Francis*, Department of Health, Government of Western Australia, Perth, Western Australia, Australia; *Belinda J Gabbe*, School of Public Health and Preventive Medicine, Monash University, Melbourne, Victoria, Australia; Health Data Research UK, Swansea University Medical School, Swansea University, Singleton Park, United Kingdom; *Adelle Gadowski*, School of Public Health and Preventive Medicine, Monash University, Melbourne, Victoria, Australia; *John Gilroy*, Aboriginal and Torres Strait Islander Research, Faculty of Medicine and Health, University of Sydney, Sydney, New South Wales, Australia; *Mitchell A Hansen,* Department of Neurosurgery, John Hunter Hospitals and University of Newcastle, Newcastle, New South Wales, Australia; *James E Harrison*, College of Medicine and Public Health, Flinders University, Adelaide, South Australia, Australia; *Luke J Haseler*, Curtin Health Innovation Research Institute, Faculty of Health Sciences, Curtin University, Perth, Western Australia, Australia; *Leanne Hassett*, Institute for Musculoskeletal Health & Sydney School of Health Sciences, Faculty of Medicine and Health, University of Sydney, Sydney, New South Wales, Australia; Sydney Local Health District, Sydney, New South Wales, Australia; *Sarah C Hellewell,* Curtin Health Innovation Research Institute & School of Medicine, Faculty of Health Sciences, Curtin University, Perth, Western Australia, Australia; Perron Institute for Neurological and Translational Science, Nedlands, Western Australia, Australia; *Amelia J Hicks*, School of Psychological Sciences, Monash University, Melbourne, Victoria, Australia; Monash Epworth Rehabilitation Research Centre, Epworth Healthcare, Melbourne, Victoria, Australia; Brain Injury Research Center, Icahn School of Medicine at Mount Sinai, New York City, New York, United States of America; *Andrew F Hill*, College of Science, Health and Engineering, La Trobe University, Melbourne, Victoria, Australia; *Andrew J A Holland*, The Children's Hospital at Westmead Clinical School, Faculty of Medicine and Health, University of Sydney, Sydney, New South Wales, Australia; *Stephen Honeybul*, Department of Neurosurgery, Sir Charles Gairdner Hospital, Perth, Western Australia, Australia; Department of Neurosurgery, Royal Perth Hospital, Perth, Western Australia, Australia; *Rosalind L Jeffree*, Kenneth G. Jamieson Department of Neurosurgery, Royal Brisbane and Women's Hospital, Brisbane, Queensland, Australia; Royal Brisbane Clinical School, School of Medicine, University of Queensland, Brisbane, Queensland, Australia; *Chris Joyce*, Intensive Care Unit, Princess Alexandra Hospital, Brisbane, Queensland, Australia; School of Medicine, University of Queensland, Brisbane, Queensland, Australia; *Elizabeth Kendall*, Menzies Health Institute Queensland, Griffith University, Brisbane, Queensland, Australia; *Kate King*, John Hunter Trauma Service, John Hunter Hospital, Newcastle, New South Wales, Australia; College of Health, Medicine and Wellbeing, University of Newcastle, Newcastle, New South Wales, Australia; *Natasha A Lannin*, Department of Neuroscience, Central Clinical School, Monash University, Melbourne, Victoria, Australia; Alfred Health, Melbourne, Victoria, Australia; *Meng Law*, Departments of Neuroscience and Radiology, Monash University, Melbourne, Victoria, Australia; Alfred Health, Melbourne, Victoria, Australia; Alzheimer's Disease Research Center & Department of Neurological Surgery, Keck School of Medicine, University of Southern California, Los Angeles, California, United States of America; *Andrew I R Maas*, Department of Neurosurgery, Antwerp University Hospital and University of Antwerp, Edegem, Belgium; *Adam Mahoney*, Trauma Service, Royal Hobart Hospital, Hobart, Tasmania, Australia; 2nd General Health Battalion, Australian Defence Force; *Peter Makin*, AUS-TBI Lived Experience Advisory Group; *Peter Mayhew,* AUS-TBI Lived Experience Advisory Group); *Alison McDonald*, AUS-TBI Lived Experience Advisory Group; *Skye McDonald*, School of Psychology, University of New South Wales, Sydney, New South Wales, Australia; *Stuart J McDonald*, Department of Neuroscience, Central Clinical School, Monash University, Melbourne, Victoria, Australia; *Ancelin McKimmie*, School of Public Health and Preventive Medicine, Monash University, Melbourne, Victoria, Australia; *Robert McNamara*, Department of Intensive Care Medicine, Royal Perth Hospital, Perth, Western Australia, Australia; School of Medicine, Faculty of Health Sciences, Curtin University, Perth, Western Australia, Australia; *Shiv Meka,* Department of Health, Government of Western Australia, Perth, Western Australia, Australia; *David K Menon*, Division of Anaesthesia, University of Cambridge, Addenbrooke's Hospital, Cambridge, United Kingdom; Wolfson Brain Imaging Centre, University of Cambridge, Cambridge, United Kingdom; *Gary Mitchell*, Emergency and Trauma Unit, Royal Brisbane and Women's Hospital, Brisbane, Queensland, Australia; Royal Brisbane Clinical Unit, University of Queensland, Brisbane, Queensland, Australia; Jamieson Trauma Institute, Brisbane, Queensland, Australia; Queensland Rugby Union; Brisbane, Queensland, Australia; *Rowena Mobbs*, Brain & Mind Centre, University of Sydney, Sydney, New South Wales, Australia; Macquarie University, Sydney, New South Wales, Australia; *Fatima A Nasrallah,* Queensland Brain Institute, University of Queensland, Brisbane, Queensland, Australia; *Virginia FJ Newcombe*, PACE Section, Department of Medicine, Addenbrooke's Hospital, University of Cambridge, Cambridge, United Kingdom; *Terence J O'Brien*, Department of Neuroscience, Central Clinical School, Monash University, Melbourne, Victoria, Australia; *John H Olver*, Epworth Healthcare, Melbourne, Victoria, Australia; Department of Medicine, Monash University, Melbourne, Victoria, Australia; *Gerard M O'Reilly*, National Trauma Research Institute, Melbourne, Victoria, Australia; Emergency and Trauma Centre, The Alfred Hospital, Melbourne, Victoria, Australia; School of Public Health and Preventive Medicine, Monash University, Melbourne, Victoria, Australia; *Tamara Ownsworth*, The Hopkins Centre, Menzies Health Institute Queensland, Griffith University, Brisbane, Queensland, Australia; School of Applied Psychology, Griffith University, Brisbane, Queensland, Australia; *Paul M Parizel*, University of Antwerp, Edegem, Belgium; Department of Radiology, Royal Perth Hospital & University of Western Australia, Perth, Western Australia, Australia; West Australian National Imaging Facility Node, Perth, Western Australia, Australia; *Michael Parr*, Intensive Care Unit, Liverpool Hospital, University of New South Wales, Sydney, New South Wales, Australia; Intensive Care Unit, Macquarie University Hospital, Macquarie University, Sydney, New South Wales, Australia; *Jennie L Ponsford*, School of Psychological Sciences, Monash University, Melbourne, Victoria, Australia; Monash Epworth Rehabilitation Research Centre, Epworth Healthcare, Melbourne, Victoria, Australia; *Bruce Powell,* AUS-TBI Lived Experience Advisory Group; *Patricia Ratajczak,* AUS-TBI Lived Experience Advisory Group; *Michael C Reade*, Faculty of Medicine, University of Queensland, Royal Brisbane and Women's Hospital, Brisbane, Queensland, Australia; Joint Health Command, Australian Defence Force, Canberra, Australian Capital Territory, Australia; *Sandy Reeder*, Department of Neuroscience, Central Clinical School, Monash University, Melbourne, Victoria, Australia; Department of Epidemiology and Preventive Medicine, School of Public Health and Preventive Medicine, Monash University, Melbourne, Victoria, Australia; *Christopher Reid*, School of Public Health, Faculty of Health Sciences, Curtin University, Perth, Western Australia, Australia; Department of Epidemiology and Preventive Medicine, School of Public Health and Preventive Medicine, Monash University, Melbourne, Victoria, Australia; *Julia Robertson*, AUS-TBI Lived Experience Advisory Group; *Suzanne Robinson*, School of Population Health, Faculty of Health Sciences, Curtin University, Perth Western Australia, Australia; *Stephen E Rose*, The Australian e-Health Research Centre, Commonwealth Scientific and Industrial Research Organisation, Brisbane, Queensland, Australia; *Jeffrey V Rosenfeld*, Department of Neurosurgery, The Alfred Hospital, Melbourne, Victoria, Australia; Department of Surgery, Monash University, Melbourne, Victoria, Australia; F. Edward Hébert School of Medicine, Uniformed Services University of The Health Sciences, Bethesda, Maryland, United States of America; *Jason P Ross*, Molecular Diagnostic Solutions, Health and Biosecurity, Commonwealth Scientific and Industrial Research Organisation, Australia; *Danette Rowse,* AUS-TBI Lived Experience Advisory Group; *Nick Rushworth*, Brain Injury Australia, Sydney, New South Wales, Australia; *Adam Scheinberg*, Neurodevelopment and Rehabilitation Research, Murdoch Children's Research Institute, Melbourne, Victoria, Australia; Department of Paediatrics, University of Melbourne, Melbourne, Victoria, Australia; *Bridgette D Semple*, Department of Neuroscience, Central Clinical School, Monash University, Melbourne, Victoria, Australia; Alfred Health, Melbourne, Victoria, Australia; Department of Medicine, Royal Melbourne Hospital, The University of Melbourne, Melbourne, Victoria, Australia; *Sandy R Shultz*, Department of Neuroscience, Central Clinical School, Monash University, Melbourne, Victoria, Australia; *Grahame K Simpson*, Brain Injury Rehabilitation Research Group, Ingham Institute for Applied Medical Research, Sydney, New South Wales, Australia; John Walsh Centre for Rehabilitation Research, Sydney School of Medicine, University of Sydney, Sydney, New South Wales, Australia; *Warwick J Teague*, Trauma Service & Department of Paediatric Surgery, The Royal Children's Hospital, Melbourne, Victoria, Australia; Surgical Research, Murdoch Children's Research Institute, Melbourne, Victoria, Australia; Department of Paediatrics, University of Melbourne, Melbourne, Victoria, Australia; *Leanne Togher*, Speech Pathology, School of Health Sciences, Faculty of Medicine and Health, University of Sydney, Sydney, New South Wales, Australia; *Andrew A Udy*, Australian and New Zealand Intensive Care Research Centre, School of Public Health and Preventive Medicine, Monash University, Melbourne, Victoria, Australia; Department of Intensive Care and Hyperbaric Medicine, The Alfred Hospital, Melbourne, Victoria, Australia; *Kirsten Vallmuur*, Centre for Healthcare Transformation, Australian Centre for Health Services Innovation, Queensland University of Technology, Brisbane, Queensland, Australia; Jamieson Trauma Institute, Brisbane, Queensland, Australia; *Dinesh Varma*, Department of Radiology, The Alfred Hospital, Melbourne, Victoria, Australia; Department of Surgery, Monash University, Melbourne, Victoria, Australia; National Trauma Research Institute, Melbourne, Victoria, Australia; *James Vickers*, Wicking Dementia Research and Education Centre, College of Health and Medicine, University of Tasmania, Hobart, Tasmania, Australia; *Janet Wagland*, Brightwater Group, Perth, Western Australia, Australia; *James Walsham*, Intensive Care Unit, Princess Alexandra Hospital, Brisbane, Queensland, Australia; School of Medicine, University of Queensland, Brisbane, Queensland, Australia; *Adam J Wells*, Department of Neurosurgery, Adelaide Hospital, Adelaide, South Australia, Australia; Department of Surgery, University of Adelaide, Adelaide, South Australia, Australia; Neurosurgical Research Foundation, Adelaide, South Australia, Australia; *Luke Whiley*, Health Futures Institute, Murdoch University, Perth, Western Australia, Australia; Perron Institute for Neurological and Translational Science, Perth, Western Australia, Australia; *Gavin Williams*, Department of Physiotherapy, Epworth Healthcare, Melbourne, Victoria, Australia; Department of Physiotherapy, University of Melbourne, Melbourne, Victoria, Australia; *Jodie K Williams*, National Critical Care and Trauma Response Centre, Royal Darwin Hospital, Darwin, Northern Territory, Australia; *Roslind Witham*, AUS-TBI Lived Experience Advisory Group; *David K Wright*, Department of Neuroscience, Central Clinical School, Monash University, Melbourne, Victoria, Australia; *Louise York*, Australian Institute of Health and Welfare, Canberra, Australian Capital Territory, Australia; *Jesse T Young*, Centre for Health Equity, Melbourne School of Population and Global Health, The University of Melbourne, Melbourne, Victoria, Australia; Centre for Adolescent Health, Murdoch Children's Research Institute, Melbourne, Victoria, Australia; School of Population and Global Health, University of Western Australia, Perth, Western Australia, Australia; National Drug Research Institute, Curtin University, Perth, Western Australia, Australia; *Heidi Zeeman*, The Hopkins Centre, Menzies Health Institute Queensland, Griffith University, Brisbane, Queensland, Australia.

## Supplementary Material

Supplementary Search Strategies

Supplementary Data Set

## Abbreviations Used

AUS-TBI: Australian Traumatic Brain Injury Initiative
CDEs: common data elements
ICD-11: International Classification of Diseases, 11th Revision
NINDS: National Institute of Neurological Disorders and Stroke
TBI: traumatic brain injury
