# Supplementary material for: The Australian Traumatic Brain Injury Initiative: Statement of Working Principles and Rapid Review of Methods to Define Data Dictionaries for Neurological Conditions
Source: Neurotrauma Rep. 2024 Apr 11;5(1):424–47. doi: 10.1089/neur.2023.0116 (PMC11040195; doi:10.1089/neur.2023.0116)
Supplement: Supplemental data [file Suppl_FileS1.docx]

Supplementary Material

In accompaniment of Bagg et al. (2023, submitted) “The Australian Traumatic Brain Injury Initiative: statement of working principles and rapid review of methods to define data dictionaries for neurological conditions”

Comprising:

1. Meta-data and search strategies (this document)

2. Paper 1_AUS-TBI_Supplementary file 2_dataset_25aug23.csv

3. Paper 1_AUS-TBI_Supplementary file 4_Investigators names and affiliations_25aug23.xlsx

#### Search strategy for MEDLINE

Search to be implemented through Ovid, for MEDLINE(R) ALL 1946 to October 26 2021

*Part A: Terms for nervous system diseases/injuries*
1 ‘nervous system’.mp or ‘nervous system’.sh
2 ‘brain’.mp or ‘brain’.sh
3 nerv.mp
4 neuro.mp
5 (traum* adj brain).mp
6 (injur* adj brain).mp
7 ‘prion’.mp or ‘Prion Diseases’.sh
8 ’glia‘.mp or ’Neuroglia’.sh
9 ‘spinal cord’.mp or ‘spinal cord’.sh
10 cerebr.mp
11 or/1-10

*Part B: Terms for common data elements*
12 ‘common data element’.mp
13 ‘Common Data Element’.sh
14 CDE.mp
15 cde.mp
16 ‘core data element’.mp
17 ‘key data element’.mp
18 ‘common knowledge element’.mp
19 ‘core knowledge element’.mp
20 ‘key knowledge element’.mp
21 ‘common information element’.mp
22 ‘core information element’.mp
23 ‘key information element’.mp
24 ‘common measurement element’.mp
25 ‘core measurement element’.mp
26 ‘key measurement element’.mp
27 ‘common evaluation element’.mp
28 ‘core evaluation element’.mp
29 ‘key evaluation element’.mp
30 ‘common data unit’.mp
31 ‘core data unit’.mp
32 ‘key data unit’.mp
33 ‘common knowledge unit’.mp
34 ‘core knowledge unit’.mp
35 ‘key knowledge unit’.mp
36 ‘common information unit’.mp
37 ‘core information unit’.mp
38 ‘key information unit’.mp
39 ‘common measurement unit’.mp
40 ‘core measurement unit’.mp
41 ‘key measurement unit’.mp
42 ‘common evaluation unit’.mp
43 ‘core evaluation unit’.mp
44 ‘key evaluation unit’.mp
45 ‘common data item’.mp
46 ‘core data item’.mp
47 ‘key data item’.mp
48 ‘common knowledge item’.mp
49 ‘core knowledge item’.mp
50 ‘key knowledge item’.mp
51 ‘common information item’.mp
52 ‘core information item’.mp
53 ‘key information item’.mp
54 ‘common measurement item’.mp
55 ‘core measurement item’.mp
56 ‘key measurement item’.mp
57 ‘common evaluation item’.mp
58 ‘core evaluation item’.mp
59 ‘key evaluation item’.mp
60 ‘biomarker’.mp
61 biomarker.sh
62 or/12-61

*Part C: Terms for methodological approaches to establishing consensus on common data elements*
63 ‘consensus’.mp or ‘consensus’.sh
64 (consensus adj based).mp
65 (consensus adj method).mp
66 (consensus adj development).mp
67 (consensus adj meeting).mp
68 (consensus adj workshop).mp
69 (consensus adj working group).mp
70 ‘consensus development conference’.sh
71 ‘consensus development’.sh
72 ‘consensus workshop’.sh
73 or/63-72

*Part D: Search results*
74 11 and 62
75 74 and 73

#### Search strategy for Embase and Emcare

Search to be implemented through Ovid, for Embase 1974 to 2021 October 27 and Emcare 1995 to 2021 Week 42

*Part A: Terms for nervous system diseases/injuries*
1 ‘nervous system’.mp or ‘nervous system’/ or ‘central nervous system’/
2 ‘brain’.mp or ‘brain’/
3 nerv.mp
4 neuro.mp
5 (traum* adj brain).mp
6 (injur* adj brain).mp
7 ‘prion’.mp or ‘prion’/ or ‘prion disease’/
8 ’glia‘.mp or ’glia’/
9 ‘spinal cord’.mp or ‘spinal cord’/
10 cerebr.mp
11 or/1-10

*Part B: Terms for common data elements*
12 ‘common data element’.mp
13 ‘Common Data Element’/
14 CDE.mp
15 cde.mp
16 ‘core data element’.mp
17 ‘key data element’.mp
18 ‘common knowledge element’.mp
19 ‘core knowledge element’.mp
20 ‘key knowledge element’.mp
21 ‘common information element’.mp
22 ‘core information element’.mp
23 ‘key information element’.mp
24 ‘common measurement element’.mp
25 ‘core measurement element’.mp
26 ‘key measurement element’.mp
27 ‘common evaluation element’.mp
28 ‘core evaluation element’.mp
29 ‘key evaluation element’.mp
30 ‘common data unit’.mp
31 ‘core data unit’.mp
32 ‘key data unit’.mp
33 ‘common knowledge unit’.mp
34 ‘core knowledge unit’.mp
35 ‘key knowledge unit’.mp
36 ‘common information unit’.mp
37 ‘core information unit’.mp
38 ‘key information unit’.mp
39 ‘common measurement unit’.mp
40 ‘core measurement unit’.mp
41 ‘key measurement unit’.mp
42 ‘common evaluation unit’.mp
43 ‘core evaluation unit’.mp
44 ‘key evaluation unit’.mp
45 ‘common data item’.mp
46 ‘core data item’.mp
47 ‘key data item’.mp
48 ‘common knowledge item’.mp
49 ‘core knowledge item’.mp
50 ‘key knowledge item’.mp
51 ‘common information item’.mp
52 ‘core information item’.mp
53 ‘key information item’.mp
54 ‘common measurement item’.mp
55 ‘core measurement item’.mp
56 ‘key measurement item’.mp
57 ‘common evaluation item’.mp
58 ‘core evaluation item’.mp
59 ‘key evaluation item’.mp
60 ‘biomarker’.mp
61 ‘biological marker’/
62 or/12-61

*Part C: Terms for methodological approaches to establishing consensus on common data elements*
63 ‘consensus’.mp or ‘consensus’/
64 (consensus adj based).mp
65 (consensus adj method).mp
66 (consensus adj development).mp
67 (consensus adj meeting).mp
68 (consensus adj workshop).mp
69 (consensus adj working group).mp
70 ‘consensus development’/
71 ‘workshop’/
72 or/63-71

73 11 and 62
74 73 and 72

#### Search strategy for PsycINFO

Search to be implemented through Ovid, for PsycINFO 1806 to October Week 4 2021

*Part A: Terms for nervous system diseases/injuries*
1 ‘nervous system’.mp or ‘nervous system’/ or ‘central nervous system’/ or ‘nervous system disorders’/
2 ‘brain’.mp or ‘brain’/ or ‘brain disorders’/ or ‘brain injuries’/
3 nerv.mp
4 ‘neuro’.mp or ’neurons’/
5 (traum adj brain).mp
6 (injur* adj brain).mp
7 ’prion’.mp or ‘prion’/
8 ’glia’.mp or ‘neuroglia’/
9 ‘spinal cord’.mp or ‘spinal cord’/
10 cerebr*.mp
11 or/1-10

*Part B: Terms for common data elements*
12 ‘common data element’.mp
13 CDE.mp
14 cde.mp
15 ‘core data element’.mp
16 ‘key data element’.mp
17 ‘common knowledge element’.mp
18 ‘core knowledge element’.mp
19 ‘key knowledge element’.mp
20 ‘common information element’.mp
21 ‘core information element’.mp
22 ‘key information element’.mp
23 ‘common measurement element’.mp
24 ‘core measurement element’.mp
25 ‘key measurement element’.mp
26 ‘common evaluation element’.mp
27 ‘core evaluation element’.mp
28 ‘key evaluation element’.mp
29 ‘common data unit’.mp
30 ‘core data unit’.mp
31 ‘key data unit’.mp
32 ‘common knowledge unit’.mp
33 ‘core knowledge unit’.mp
34 ‘key knowledge unit’.mp
35 ‘common information unit’.mp
36 ‘core information unit’.mp
37 ‘key information unit’.mp
38 ‘common measurement unit’.mp
39 ‘core measurement unit’.mp
40 ‘key measurement unit’.mp
41 ‘common evaluation unit’.mp
42 ‘core evaluation unit’.mp
43 ‘key evaluation unit’.mp
44 ‘common data item’.mp
45 ‘core data item’.mp
46 ‘key data item’.mp
47 ‘common knowledge item’.mp
48 ‘core knowledge item’.mp
49 ‘key knowledge item’.mp
50 ‘common information item’.mp
51 ‘core information item’.mp
52 ‘key information item’.mp
53 ‘common measurement item’.mp
54 ‘core measurement item’.mp
55 ‘key measurement item’.mp
56 ‘common evaluation item’.mp
57 ‘core evaluation item’.mp
58 ‘key evaluation item’.mp
59 ‘biomarker’.mp
60 ’biological markers*’/
61 or/12-60

*Part C: Terms for methodological approaches to establishing consensus on common data elements*
62 ‘consensus’.mp
63 (consensus adj based).mp
64 (consensus adj method).mp
65 (consensus adj development).mp
66 (consensus adj meeting).mp
67 (consensus adj workshop).mp
68 (consensus adj working group).mp
69 ‘group decision making’/
70 ‘group discussion’/
71 or/62-70

72 11 and 61
73 72 and 71

#### Search strategy for CINAHL

Search implemented through EBSCOHost, for CINAHL xxx to xxx Week x 2021

*Part A: Terms for nervous system diseases/injuries*
S1 nervous system or (MH “nervous system’)
S2 brain or (MH”brain”)
S3 nerv*
S4 neuro*
S5 traum* W5 brain
S6 injur* W5 brain
S7 prion or (MH “Prion Diseases”)
S8 glia* or (MH “Neuroglia”)
S9 spinal cord or (MH “spinal cord”)
S10 cerebr*
S11 S1 or S2 or S3 or S4 or S5 or S6 or S7 or S8 or S9 or S10

*Part B: Terms for common data elements*
S12 common data element
S13 (MH “Common Data Element”)
S14 CDE
S15 cde
S16 core data element
S17 key data element
S18 common knowledge element
S19 core knowledge element
S20 key knowledge element
S21 common information element
S22 core information element
S23 key information element
S24 common measurement element
S25 core measurement element
S26 key measurement element
S27 common evaluation element
S28 core evaluation element
S29 key evaluation element
S30 common data unit
S31 core data unit
S32 key data unit
S33 common knowledge unit
S34 core knowledge unit
S35 key knowledge unit
S36 common information unit
S37 core information unit
S38 key information unit
S39 common measurement unit
S40 core measurement unit
S41 key measurement unit
S42 common evaluation unit
S43 core evaluation unit
S44 key evaluation unit
S45 common data item
S46 core data item
S47 key data item
S48 common knowledge item
S49 core knowledge item
S50 key knowledge item
S51 common information item
S52 core information item
S53 key information item
S54 common measurement item
S55 core measurement item
S56 key measurement item
S57 common evaluation item
S58 core evaluation item
S59 key evaluation item
S60 biomarker
S61 (MH “biomarker”)
S62 S12 or S13 or S14 or S15 or S16 or S17 or S18 or S19 or S20 or S21 or S22 or S23 or S24 or S25 or S26 or S27 or S28 or S29 or S30 or S31 or S32 or S33 or S34 or S35 or S36 or S37 or S38 or S39 or S40 or S41 or S42 or S43 or S44 or S45 or S46 or S47 or S48 or S49 or S50 or S51 or S52 or S53 or S54 or S55 or S56 or S57 or S58 or S59 or S60 or S61

*Part C: Terms for methodological approaches to establishing consensus on common data elements*
S63 consensus or (MH “consensus”)
S64 consensus W3 based
S65 consensus W3 method
S66 consensus W3 development
S67 consensus W3 meeting
S68 consensus W3 workshop
S69 consensus W3 working group
S70 (MH “consensus development conference”)
S71 (MH “consensus development”)
S72 (MH “consensus workshop”)
S73 S63 or S64 or S65 or S66 or S67 or S68 or S69 or S70 or S71 or S72

*Part D: Search results*
S74 S11 and S62
S75 S74 and S73
